# Restoration of clean water supply and toilet hygiene reduces infectious diseases in post-disaster evacuation shelters: A multicenter observational study

**DOI:** 10.1016/j.heliyon.2021.e07044

**Published:** 2021-05-14

**Authors:** Tetsuya Akaishi, Kazuma Morino, Yoshikazu Maruyama, Satoru Ishibashi, Shin Takayama, Michiaki Abe, Takeshi Kanno, Yasunori Tadano, Tadashi Ishii

**Affiliations:** a Department of Education and Support for Regional Medicine, Tohoku University, Sendai, Japan; b Department of Emergency Medicine, Yamagata Prefectural Central Hospital, Yamagata, Japan; c Department of Disaster Medicine, Japan Red Cross Medical Center, Tokyo, Japan; d Department of Emergency Medicine, Japan Red Cross Ishinomaki Hospital, Ishinomaki, Japan

**Keywords:** Disaster, Evacuation shelter, Infectious diseases, Toilet hygiene, Water supply

## Abstract

After a massive disaster, many residents in affected areas are forced to temporarily stay in evacuation shelters. The exact impact of the state of resource supply and infrastructure in evacuation shelters on the health status of evacuees has not been sufficiently studied. Two weeks after the 2011 Great East Japan Earthquake (GEJE), comprehensive surveillance related to the health status and hygiene level was performed for all evacuation shelters (328 shelters with 46,480 evacuees at the peak) in one of the most devastating medical zones after the tsunami hit the area (Ishinomaki City). The joint relief team regularly visited all evacuation shelters across the area to assess the situation of resource supply levels, infrastructural damage, rapid need of resources, and the health status of the evacuees. In this cross-sectional observational study, we evaluated the relationship between the resource supply levels and health status among evacuees in two time periods (days 14–19 and 20–25). Among the evaluated vital resources, clean tap water supply was among the most disrupted by the disaster, and was not fully restored in most shelters during the assessment period. The cross-sectional relationship between resource supplies and morbidity was inconsistent between the two assessment periods, reflecting the multifactorial nature of health status in evacuation shelters. The clean tap water supply level at the first assessment showed a strong negative correlation with the subsequent prevalence of respiratory or gastrointestinal infectious conditions at the second assessment. Restorations in the clean tap water supply and toilet hygiene correlated each other, and both correlated with a decrease in the prevalence of gastrointestinal infectious conditions. In conclusion, disrupted clean tap water supply and inadequate toilet hygiene after a massive disaster would jointly harm the health status of those in shelters. Prompt assessments using quick visual assessment and restorations of these key resources have validity with suppressed environmental health risks among evacuees.

## 1 Introduction

After a massive catastrophic disaster, humanitarian actions are needed to protect life and health of disaster victims, with dignity, comfort and security [1]. After a catastrophic natural disaster like and earthquake with tsunamis, many survivors from all socioeconomic categories are forced to evacuate to non-home-like conditions, like evacuation shelters [2]. Such survivors are known to suffer from many mental and physical disturbances during their mid-to-long term displacement [3, 4]. Previously, shelter surveillance to assess the level of hygiene, conduct rapid need assessment, and assess the health status of evacuees after massive disasters has been performed worldwide [5, 6, 7], like when the 2005 Hurricane Katrina struck Louisiana. Close contact among evacuees in unhygienic conditions without sufficient ventilation have increased the environmental health risks, including respiratory, gastrointestinal, and skin infections [8, 9]. For example, the outbreak of norovirus among shelter evacuees in a large stadium in Houston occurred after hurricane Katrina [10, 11, 12]. The importance of mental healthcare for post-disaster evacuees has also been reported [13, 14]. More recently, possible relationships between disaster-derived stress and cardiovascular, renal, or metabolic diseases have been reported [15, 16, 17]. To suppress such environmental health risks among evacuees, resource supplies and sanitation are known to be critical determinants as an inextricable human right [1]. Based on accumulated experience and knowledge, several guidelines have been developed in the humanitarian sector, such as the Sphere Project, Active Learning Network for Accountability and Performance in Humanitarian Action (ALNAP), and Humanitarian Accountability Partnership (HAP), to standardize humanitarian aid activities in response to natural and manmade disasters [1, 18, 19]. However, there are indications that the present standards in the key lifesaving humanitarian aid sector, including water supply, sanitation and hygiene promotion, and food security, need further investigation and evidence-based verification for the association between humanitarian intervention and health outcome [20, 21]. Moreover, the short-to medium-term impact of resource supply levels and infrastructural damage of post-disaster evacuation shelters on the physical health of the evacuees—such as the prevalence of common physical symptoms—has yet to be thoroughly studied.

On May 11, 2011, the Great East Japan Earthquake (GEJE), with a magnitude of 9.0, hit Japan; subsequently, it was followed by a massive tsunami along the wide coastal areas on the Pacific side [22]. The height of the waves reached higher than 33 feet (10 m), and the run-up height of the tsunami reached close to 130 feet (40 m) [23]. Furthermore, most of the coastal cities on the Pacific side of Tohoku region (the northern part of Japan's main island) were severely damaged, rendering a tremendous number of the residents dead or missing. At the end of March 2021, the death toll was more than 15,000 (drowning accounted for about 90% of the deaths) [24, 25], and more than 2,500 are still missing. Together with the subsequent triple nuclear meltdown at the Fukushima Daiichi Nuclear Power Plant, it took several weeks after the earthquake to accurately grasp the whole picture of the tragic damage in the disaster area [26]. Because the transportation system in the Tohoku region was almost completely stopped for several weeks, nearly all the residents in Iwate, Miyagi, and Fukushima Prefectures (i.e., the Pacific side of Tohoku District), with a combined population of more than five million, were temporarily isolated without sufficient resource supplies. This is one of the largest natural disasters in global history as it directly hit an advanced country and caused numerous casualties [25]. In addition to the aforementioned casualties and missing people, nearly 4,000 people were deceased by disaster-related deaths during sheltering or evacuations. At the peak, three days after the earthquake, nearly 500,000 people were evacuated to public or non-public shelters across the nation [27], and it took nearly a year after the GEJE to close every evacuation shelter. After the disaster, many evacuees living in non-home-like conditions suffered from limited access to resources (such as food and water) and insufficient distribution of the supplied resources for a long time, facing environmental health risks that affected survival and healthy life [28].

The Ishinomaki Medical Zone, composed of Ishinomaki, Higashimatsushima, and Onagawa, in Miyagi Prefecture, was one of the areas most severely damaged by the tsunami. There were 5,385 casualties and 710 people were missing in the Ishinomaki Medical Zone, which had a population of approximately 220,000 at the time of the GEJE. The Ishinomaki Zone Joint Relief Team (IZJRT) was established to survey and support the disaster victims in the medical zone on day 10 and functioned as the local front headquarters of the medical zone. The team was headed by the last author of this article (TI), who was then the Miyagi Prefecture disaster medical coordinator in charge of the Ishinomaki area. The joint relief team had oversight functions of the medical staff and disaster medical assistance teams (DMATs); volunteers were enlisted from other areas, and medical resources were delivered to the medical zone [29]. There was a need to swiftly and correctly comprehend the overview of the incurred damage to the resources and infrastructure as well as the conditions of public and non-public evacuation shelters, many of which were not officially recognized or supported soon after the disaster.

In this two-timepoint cross-sectional multicenter observational study, by using the original raw data of the shelter assessments collected from repeated field surveillance by the IZJRT, we analyzed the association between the resource supply or hygiene level and the subsequent health situation in each shelter. We focused on mid-to-large-sized evacuation shelters with ≥50 accommodated evacuees. We then analyzed the data to elucidate the impact of resource supply levels in evacuation shelters in relation to the health status of the evacuees.

## 2 Materials and methods

### 2.1 Study design

**Figure 1:**
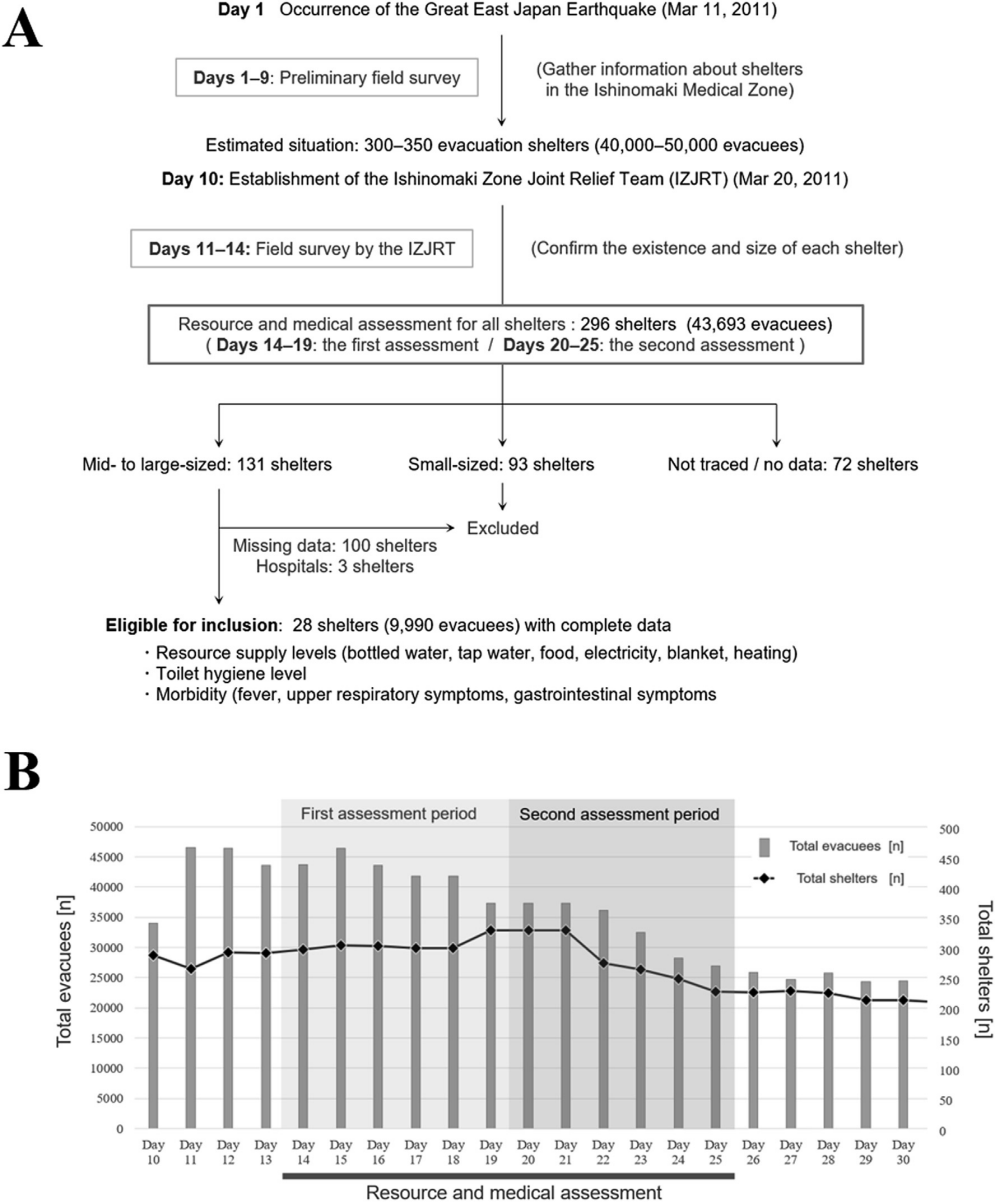
Flow diagram of enrollment for resource assessments and medical checkups. (A) Based on the information of the preliminary field survey, more than 300 evacuation shelters were inspected by the IZJRT regarding resource supply levels, infrastructural damage, and the health status of the evacuees. More than half of the shelters were mid-to-large-sized with ≥50 accommodated evacuees. A total of 28 mid-to-large-sized shelters with 9,990 evacuees were cross-sectionally assessed for the above-described modalities. (B) The timing of the resource assessments and medical checkups due to the chronological changes in the number of total shelters and accommodated evacuees in the Ishinomaki Medical Zone (total population of 220,000).

The flow diagram of the enrollment in this project is shown in Figure 1A. After the GEJE, Miyagi Prefecture and local headquarters jointly started collecting information on public and non-public evacuation shelters scattered across the Ishinomaki Medical Zone. By day 10, more than 300 shelters, with about 40,000 to 50,000 evacuees, were estimated to exist based on preliminary field surveys by municipal governments. Based on the preliminary data about shelter locations, doctors and other medical staff regularly and collaboratively visited all of them. From days 7–9, surveys were carried out by medical staff from the Japanese Red Cross Ishinomaki Hospital, who were supported by relief teams from the Japanese Red Cross Society dispatched from other parts of the country. The members visited all the shelters and confirmed their existence, the resource supply levels, infrastructural damage, and the health of the evacuees. After the establishment of the IZJRT on day 10, the survey was continued in order to update the data, along with regular visits to all the shelters; subsequently, the data were saved chronologically. During the assessment period, 40–60 relief teams participated daily. The number of visited shelters peaked at 328 (day 19), and the number of assessed evacuees peaked at 45,000–50,000 (day 11). Specific numbers of accommodated evacuees were available for 224 shelters (29,847 evacuees in total), of which 131 were mid-to-large-sized with ≥50 accommodated evacuees (27,770 total). The remaining 93 shelters were small, with <50 accommodated evacuees (2,077 in total). The chronological changes in the total number of visited shelters and assessed evacuees in the Ishinomaki Medical Zone by the IZJRT, together with the timing of the resource assessment periods and medical checkups, are shown in Figure 1B. An original uniform paper-based assessment sheet was completed. The sheet included items to check for resource supply levels, rapid need, shelter hygiene situation, existence of evacuees with chronic physical or mental conditions, and prevalence of infectious conditions (fever, respiratory symptoms, gastrointestinal symptoms) in each shelter. The assessment was performed repeatedly during the assessment period (days 14–25) of this study, in each shelter. Shelters without missing data in the first (days 14–19) and second assessment periods (days 20–25) were considered eligible for the subsequent statistical analyses.

Small-sized shelters with <50 evacuees were excluded from the analyses because most of these small shelters were relocated or closed during the assessment period. Furthermore, such small shelters with small denominators may produce shelter size-based biases when calculating the morbidity rate in each shelter. Among the 131 mid-to-large-sized shelters with ≥50 evacuees, three (with 230, 300, and 176 evacuees, respectively) were located within hospitals and were not regularly followed because of the abundance of resource supplies and medical backups. The remaining 128 mid-to-large-sized shelters with regular visits by team members were initially recruited for this study. In these initially recruited 128 shelters, 100 had missing essential data on either the first or second assessments, making them ineligible for subsequent statistical analyses. In detail, three of the ineligible 100 shelters were closed during the assessment period and assessment data were completely missing in the second assessment; the remaining 97 shelters were ineligible because of incomplete datasets of at least one blank column on key variables of resource supply and/or health status. Consequently, assessment data from the remaining 28 shelters with 9,990 accommodated evacuees were eligible and enrolled in the following statistical analyses.

**Figure 2:**
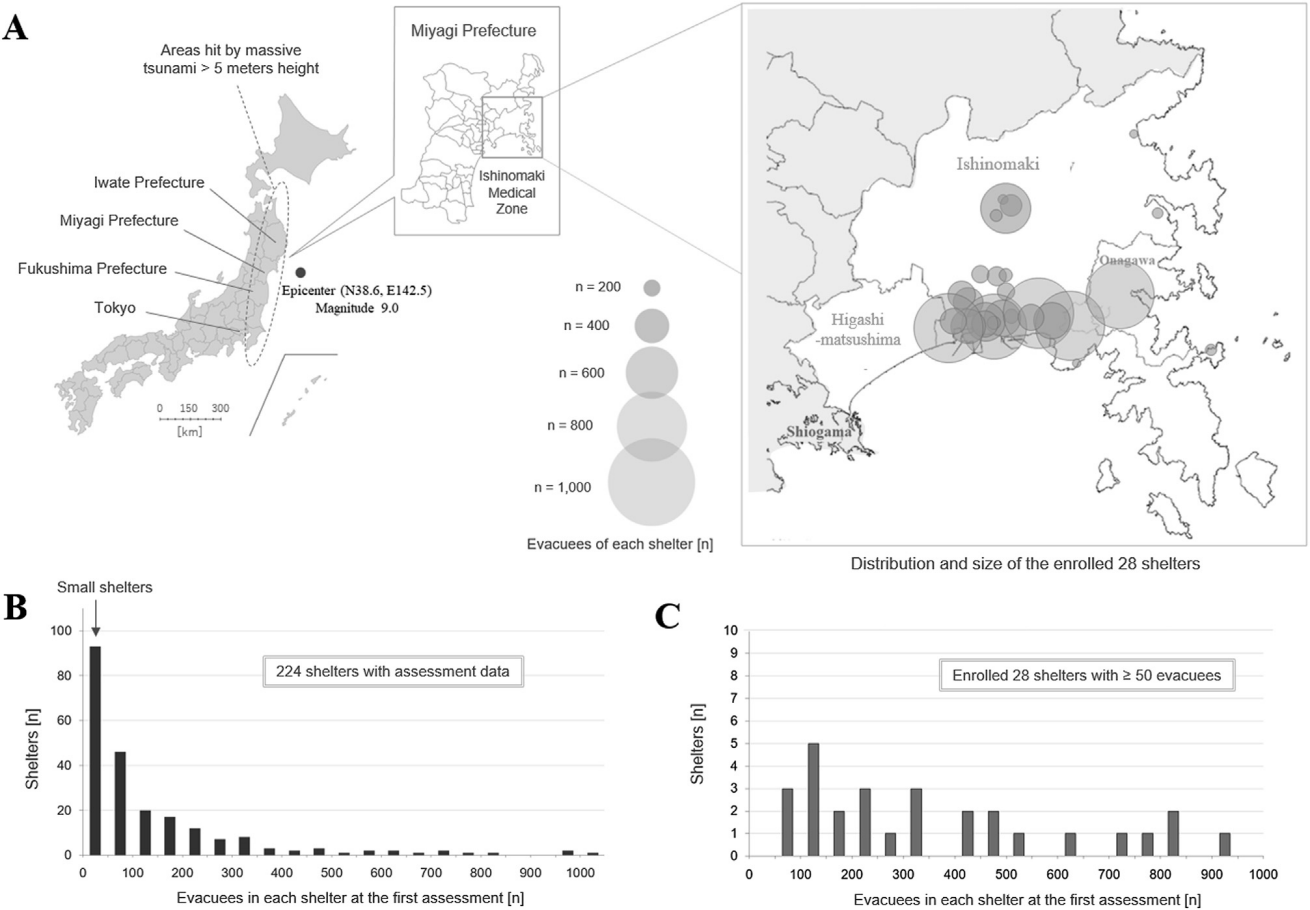
Geographic distribution and population size of the assessed shelters. (A) Geographic locations of the assessed 28 evacuation shelters in the Ishinomaki Medical Zone, one of the nearest cities from the epicenter of the earthquake. (B) A histogram of the population size of all public and non-public evacuation shelters established in the medical zone, confirmed by the initial visiting from day 11 to day 14. More than half of the shelters were mid-to-large-sized with the population size ≥50. (C) A histogram of the assessed 28 shelters with complete assessment data concerning the resource supply levels, infrastructural damage, and health statuses.

**Figure 3:**
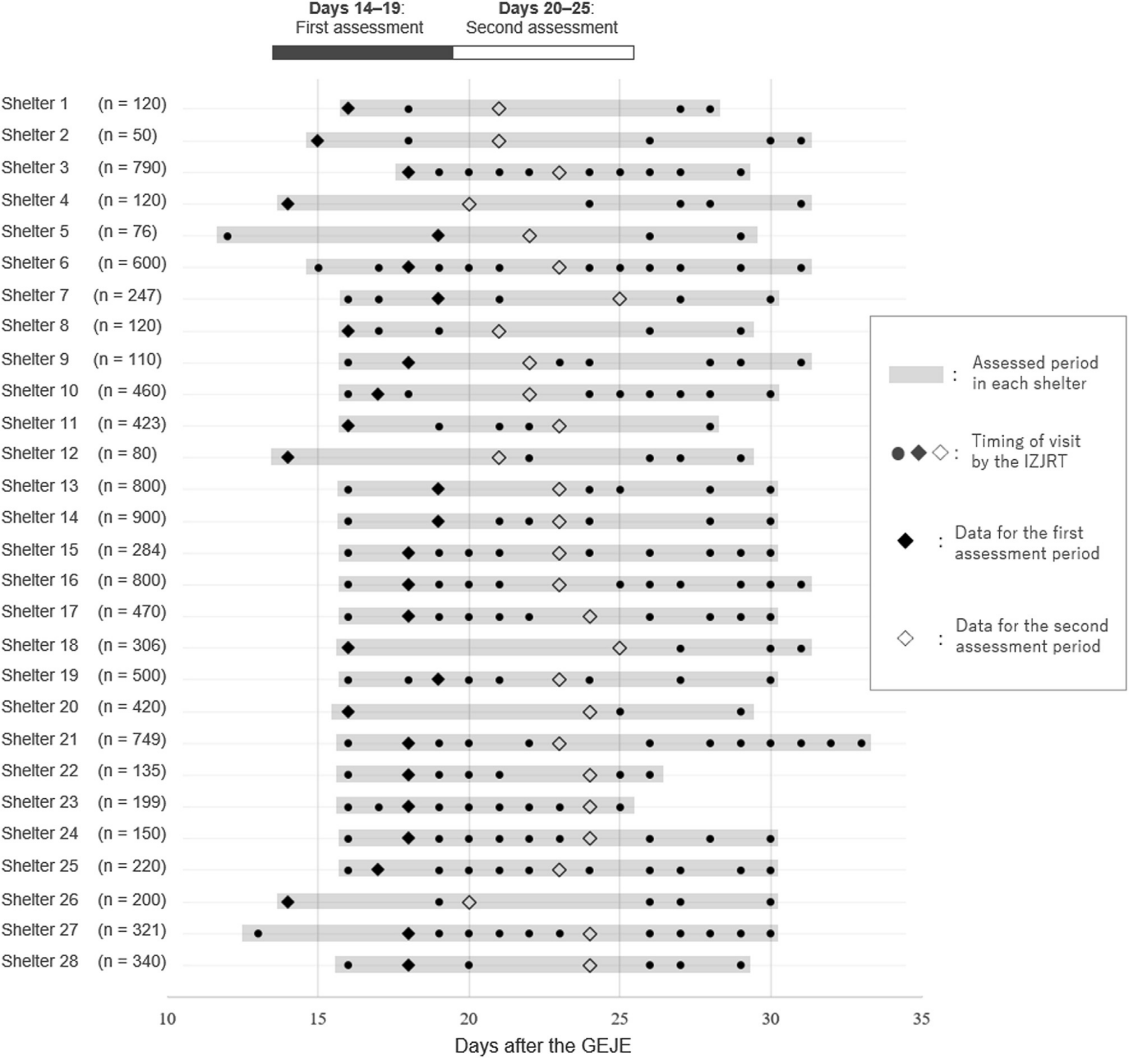
The timing of the visits, cross-sectional data, and follow-up data for each shelter. The displayed numbers aside from the shelter numbers are the reported number of accommodated evacuees in each shelter at the time of the first cross-sectional data sampling (black diamond). IZJRT, Ishinomaki Zone Joint Relief Team; GEJE, Great East Japan Earthquake.

The geographical distribution of the 28 shelters is shown in Figure 2A. Most of the shelters were located on the coastal side, which was devastated by the tsunami. The four shelters located inland at the center of Ishinomaki were established along the Kitakami River, through which the tsunami traveled upstream as far as 50 km from the estuary, leading to many casualties in the riverside area [22, 30]. These shelters, across the Kitakami River, also accommodated many evacuees from the devastated coastal areas. The histograms of the population size in each shelter among the 224 and the 28 finally enrolled shelters are shown in Figure 2B and C. The visiting time and the timing of the cross-sectional and follow-up data collection for each of the 28 shelters are shown in Figure 3.

### 2.2 Evaluated variables

From the 28 enrolled shelters with complete assessment data on the resource supplies and health situation, data on resource supply level, infrastructural damage, and prevalence of common diseases were comprehensively evaluated between days 14 and 25 (28 shelters; 9,990 evacuees). For the cross-sectional analysis, data collected between days 14 and 19 (first cross-sectional assessment) and 20 and 25 (follow-up assessment) were used. These two paired datasets were subsequently used for the longitudinal analysis to evaluate the relationship between the resource supply level and health status among evacuees in each shelter.

**Figure 4:**
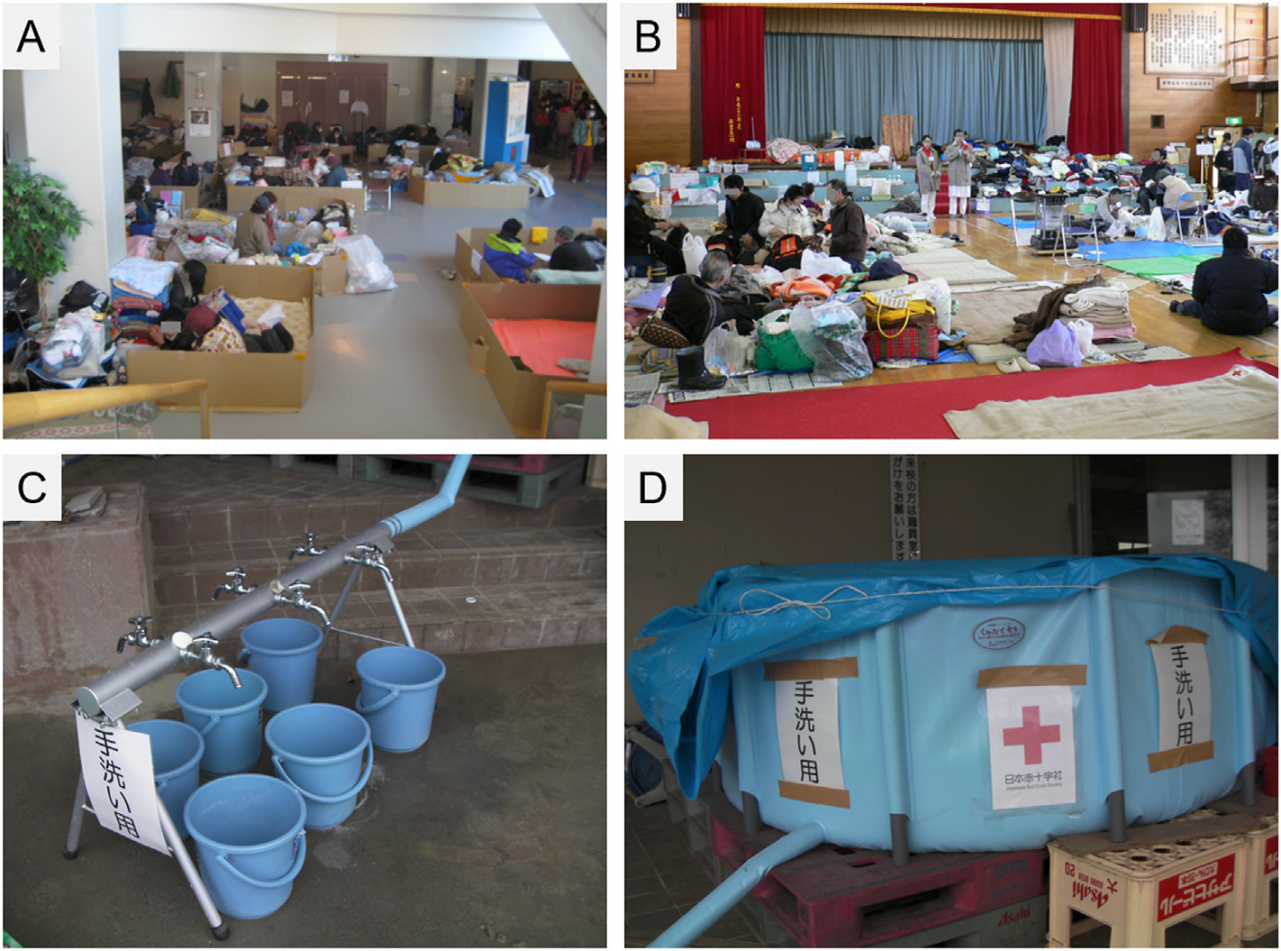
Pictures of the evacuation shelters during the first assessment period. (A) A scene of a large shelter with ≥500 evacuees. This shelter was one of the most supplied and sanitized shelters with low morbidity among the evacuees. (B) A scene of a medium-sized shelter with about 100 evacuees. (C, D) Temporary emergent tap water supply facility with a water tank and six faucets. This case was assessed as “sufficient”.

**Figure 5:**
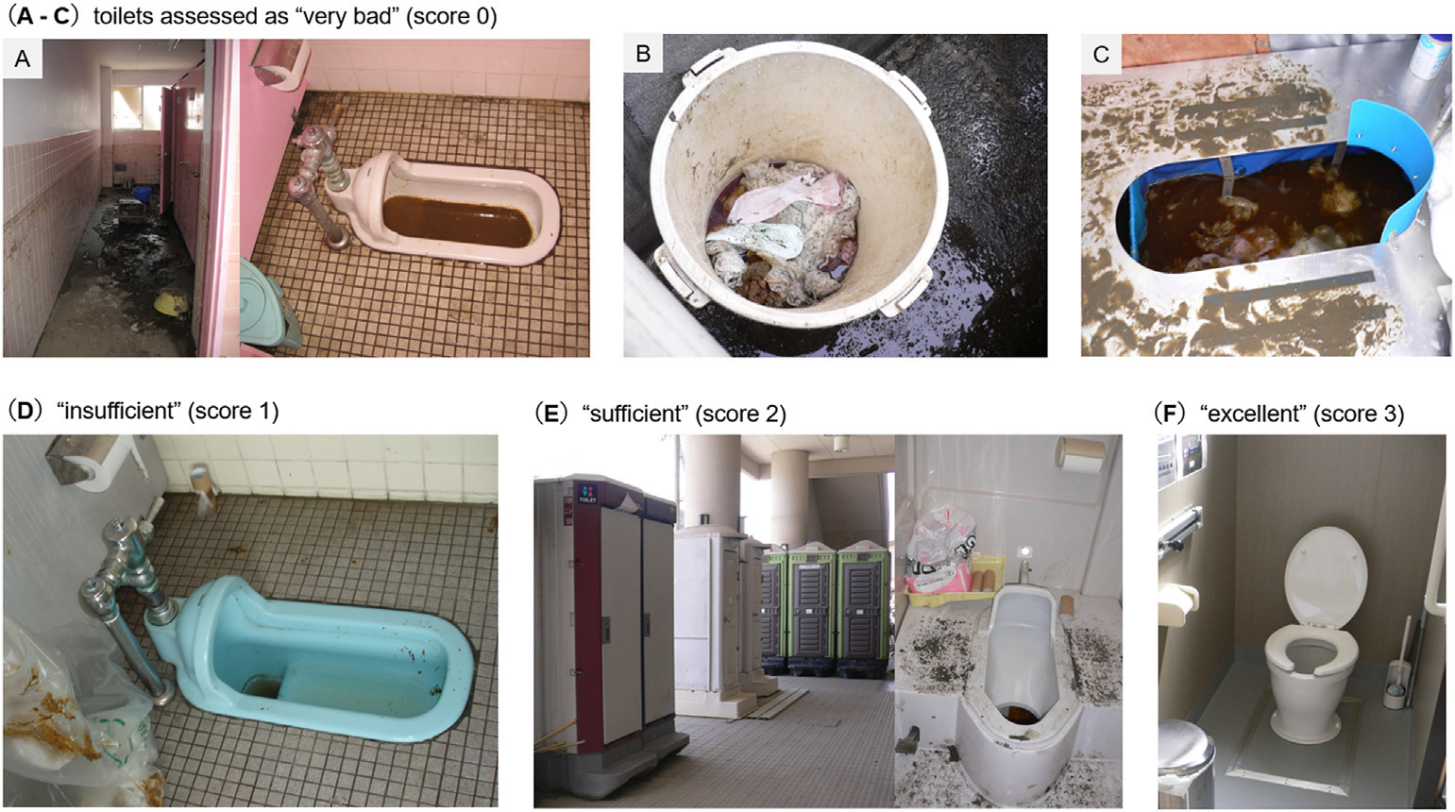
Pictures of toilets in the shelters during and after the first assessment period. (A–E) Pictures of the actual toilet scenes during the first assessment period (days 14–19), taken by the assessment team members. Regularly cleaned and desludged temporary toilet with hand washing facility was assessed as “sufficient”. (F) A picture of the undamaged functional toilet in an evacuation shelter, taken after the second assessment period in 2011. Only the toilets of such undamaged fixed toilets were assessed as “excellent”.

The resource supply levels were evaluated based on the following factors: bottled water for drinking, clean tap water, food, electricity, blankets, heating, and toilet hygiene levels in each facility, obtained by the direct interviews with the evacuees, followed by reconfirmation by the relief team at each visit. The supply levels were visually assessed by the assessment team members and scored as 0 (none/very bad), 1 (insufficient), 2 (sufficient), or 3 (undamaged/excellent). As for the infrastructural resources (tap water, electricity, toilet), undamaged or fully restored original resources were assessed as “excellent.” If temporary emergent equipment was supplied to substitute the damaged original resources, the resource level was assessed as “sufficient” or “insufficient,” based on whether they met the minimum daily requirement to secure a healthy life with comfort and low environmental health risks. The assessed tap water supply included water from trucks or water tanks provided by the local government or the Japan Self-Defense Forces. The actual scenes of the evacuation shelters and temporary water supply facility (assessed as “sufficient”) during the first assessment period, are shown in Figure 4. The actual scenes of the assessed toilets in the evacuation shelters during and after the assessment periods are shown in Figure 5. Shelters with heavily contaminated unfunctional toilets without useable temporary emergency toilets were assessed as “none/very bad” (Figure 5A–C). Relatively clean unfunctional (i.e., cannot flush water) original toilets that required waste disposal after each use by the user was assessed as “insufficient” (Figure 5D). Temporary emergency toilets that were ill-managed (contaminated, no regular desludge) were also assessed as “insufficient.” Temporary emergent toilet with regular desludge and cleaning, equipped with hand washing facility, was assessed as “sufficient” (Figure 5E).

The health status of each shelter was evaluated by the total number and the percentage of the following patients: those who required medical examination with or without prescriptions, those with a fever ≥38.0 °C, those with upper respiratory symptoms (URS), and those with gastrointestinal symptoms (GIS).

For statistical convenience, changes (Δ) in the morbidity rate (%) and resource supply score (0–3) between the first and second assessments were substituted by the subtraction of the values as follows:

$${\text{Δ}}_{resource}=\;\left(resource\;score\;at\;the\;second\;assess\right)-\;\left(score\;at\;the\;first\;assess\right)$$

$${\text{Δ}}_{morbidity}=\;\left(disease\;prevalence\;at\;the\;second\;assess\right)-\;\left(prevalence\;at\;the\;first\;assess\right)$$

Data cleaning and the collection of several additional data concerning the shelter environment, such as shelter floor size and population density, continued until April 2020.

### 2.3 Statistical analysis

Distributions of resource supply and disease prevalence from the 28 enrolled shelters are described as the median and interquartile range (IQR; 25–75 percentile). Comparisons of two paired variables between the first and second assessments were performed using the Wilcoxon signed-rank test, based on the non-normal distributions of most of the evaluated variables. The correlation between two variables with non-normal distributions was evaluated using Spearman's correlation coefficient (rho), followed by the test of no correlation. The correlation between two variables with non-normal distributions after adjusting for the influence of a third covariant factor was evaluated by calculating the non-parametric partial correlation coefficient while controlling for the covariates. Because multiple pairs of variables were simultaneously evaluated in each assessment theme, a p-value of less than 0.01, was considered statistically significant in this study. We used SPSS Statistics Base 22 (IBM Corp., Armonk, NY, USA), and MATLAB R2015a (MathWorks).

### 2.4 Ethical approval

The study was approved by the Institutional Review Board of the Tohoku University Graduate School of Medicine (IRB approval number: 2020-1-372). The Institutional Review Board waived the requirement for written informed consent from the participants because this study was not an individual-based survey and because of the urgency to collect necessary massive data just after the occurrence of the disaster. Informed consent was secured in an opt-out manner.

## 3 Results

### 3.1 Summary of the resource and medical assessment data

**Figure 6:**
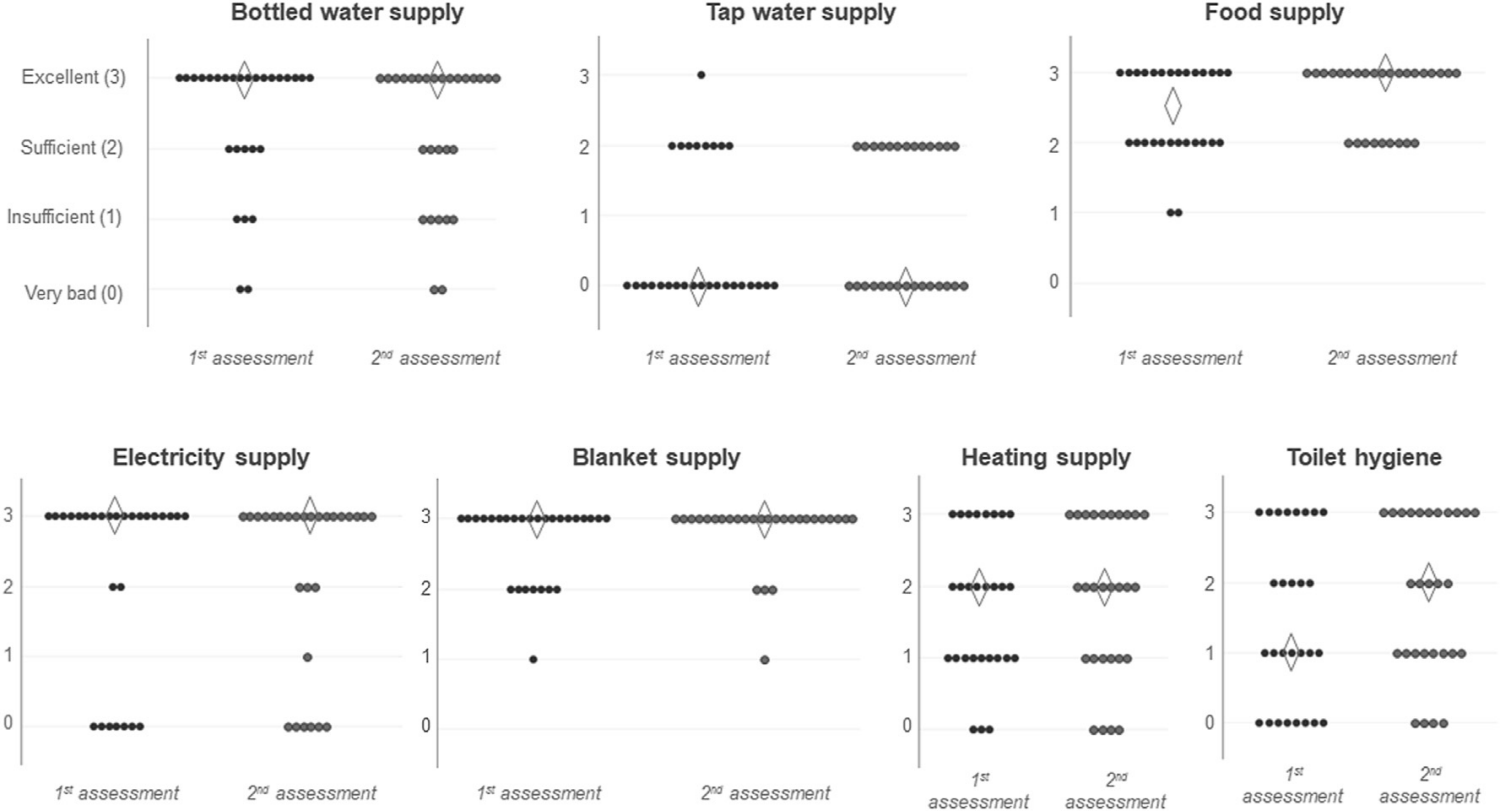
Distributions of the assessed resource supply levels in each shelter. Each plot shows the assessed level of resource supply in each shelter. The diamonds show the medians of the distributions.

The summarized cross-sectional data on resource supply levels and the prevalence of the studied symptoms among the enrolled 28 shelters are shown in Table 1. The number of evacuees accommodated in each shelter decreased from the beginning to the end of the assessment period (p = 0.0002, Wilcoxon signed-rank test). The shelter floor area that could be freely used by evacuees in each of the 28 shelters was 5,562 ± 2,615 m^2^. The distributions of each resource supply level in the first and second assessments from the enrolled 28 shelters are shown in Figure 6. The supply levels of all evaluated resources showed apparent non-normal distributions. The clean tap water supply was the most severely damaged resource in both first and second assessment periods, without significant restoration between the periods in total. The raw data for enabling the reproducibility and verification of the subsequent analyses are shown in Supplementary Table 1. To clarify the concurrent relationship between shelter size and the studied variables, the Spearman's correlation coefficients (rho) between the shelter size-related variables (e.g., population size, shelter width, and population density) and the simultaneous resource supply or health status data, during the first assessment period, were calculated (Table 2). None of the assessed resource supply levels or health statuses was significantly affected by the shelter size, except for a moderate negative correlation between the population size and the rate of all patients.

**Table 1:** Summary of the cross-sectional resources and medical assessment data.

	At the first cross-sectional assessment (days 14–19)	At the second cross-sectional assessment (days 20–25)	p-values
Assessed shelters and accommodated evacuees in total			
Total shelters (n)	296 shelters	328 shelters	-
Total evacuees (n)	43,693	37,289	-
Population size of the eligible 28 shelters with complete assessment data			
Total evacuees (n)	9,990	8,706	-
Evacuees in each shelter (n)^a^	295 (131–478)	240 (107–432)	<0.001∗
Resource supply status^a^^,^^b^			
Bottled water supply (0–3)	3.0 (2.0–3.0)	3.0 (2.0–3.0)	0.624
Tap water supply (0–3)	0.0 (0.0–2.0)	0.0 (0.0–2.0)	0.080
Food supply (0–3)	2.5 (2.0–3.0)	3.0 (2.0–3.0)	0.100
Electricity supply (0–3)	3.0 (2.0–3.0)	3.0 (2.0–3.0)	1.000
Blanket supply (0–3)	3.0 (2.5–3.0)	3.0 (3.0–3.0)	0.208
Heating supply (0–3)	2.0 (1.0–3.0)	2.0 (1.0–3.0)	0.529
Hygiene of toilet (0–3)	1.5 (1.0–3.0)	2.0 (1.0–3.0)	0.164
Morbidity a			
Total patients (%)	13.5% (9.8–22.0)	11.4% (7.0–18.5)	0.062
Patients with fevers ≥38.0 °C (%)	0.3% (0.0–0.8)	0.1% (0.0–0.4)	0.085
Patients with URS (%)	4.9% (3.4–10.8)	3.3% (2.5–10.7)	0.062
Patients with GIS (%)	0.8% (0.3–2.1)	0.5% (0.0–1.2)	0.013

All the paired comparisons between the first and second assessment periods were performed by the Wilcoxon's signed rank test.

GIS, gastrointestinal symptoms; URS, upper respiratory symptoms.

a Median and interquartile range (25–75 percentiles).

b The resource supply levels were visually assessed by the assessment team members and scored using “0” (“none/very bad”), “1” (“insufficient”), “2” (“sufficient”), and “3” (“undamaged/excellent”).

**Table 2:** Correlation coefficients (rho) between the shelter size and concurrent data at the first cross-sectional assessment (days 14–19).

	Population-based data (average during assessment period)		
	Population size [n]	Shelter width [m^2^]	Population density [n/m^2^]
Bottled water (0–3)	0.029 (p = 0.883)	−0.210 (p = 0.283)	0.195 (p = 0.319)
Tap water (0–3)	0.404 (p = 0.033)	0.245 (p = 0.210)	0.187 (p = 0.340)
Food (0–3)	−0.302 (p = 0.118)	−0.236 (p = 0.227)	−0.121 (p = 0.538)
Electricity (0–3)	0.195 (p = 0.319)	0.280 (p = 0.149)	−0.198 (p = 0.312)
Blanket (0–3)	−0.064 (p = 0.745)	−0.128 (p = 0.517)	0.155 (p = 0.431)
Heating (0–3)	−0.339 (p = 0.078)	−0.342 (p = 0.075)	−0.012 (p = 0.952)
Toilet hygiene (0–3)	0.135 (p = 0.493)	−0.031 (p = 0.875)	0.352 (p = 0.066)
Total patients (%)	−0.488 (p = 0.008) ∗	−0.305 (p = 0.115)	−0.299 (p = 0.122)
Fever ≥38.0 °C (%)	−0.159 (p = 0.421)	−0.002 (p = 0.991)	−0.361 (p = 0.059)
URS (%)	−0.374 (p = 0.050)	−0.408 (p = 0.031)	0.007 (p = 0.971)
GIS (%)	0.001 (p = 0.997)	0.262 (p = 0.178)	−0.309 (p = 0.110)

GIS, gastrointestinal symptoms; URS, upper respiratory symptoms.

Spearman's correlation coefficients (rho) between the population size and the assessed variables are shown in this correlation matrix. The shown p-value aside of each correlation coefficient is the result of the test of no correlation.

∗p < 0.01.

### 3.2 Simultaneous impact of resource supply levels on health status

**Figure 7:**
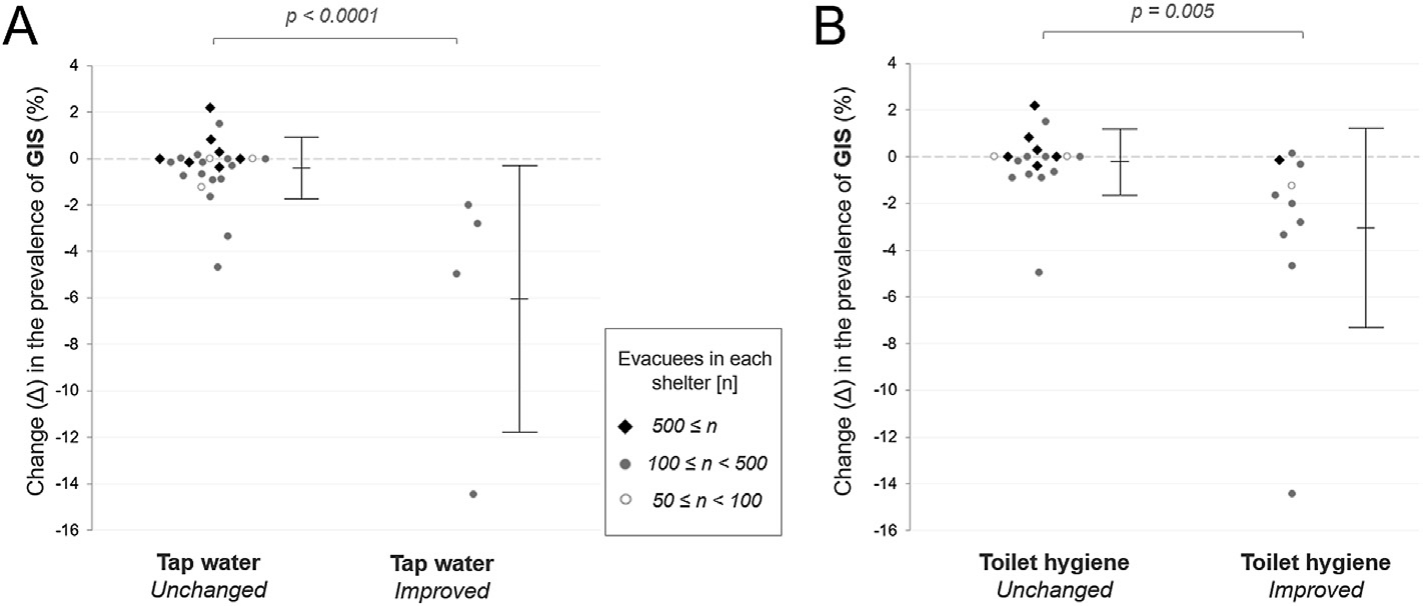
Prevalence of GIS by the recovery of resource supply levels. (A) The prevalence of GIS was significantly lower in shelters with improved tap water supply. (B) The prevalence of GIS was also significantly lower in shelters with improved toilet hygiene. GIS, gastrointestinal symptoms.

To clarify the cross-sectional concurrent relationship between the resource supply levels and the health situation in each shelter, we calculated the Spearman's correlation coefficients (rho) for all pairs of the two modalities at the initial assessment period (first half of Table 3). During the first assessment period, the level of toilet hygiene significantly influenced the concurrent prevalence of GIS (rho = −0.51, p = 0.0057). A cross-sectional analysis of the subsequent second assessment data (i.e., days 20–25) was also performed, the results of which are shown in the second half of Table 3. During the second assessment period, the supply levels of bottled water, tap water, electricity, and heating showed significant negative correlations with at least one of the medical assessment data. Next, to clarify the longitudinal mutual relationship between the resource supply level and the health situation in each shelter, we calculated the Spearman's correlation coefficients (rho) between the changes after the follow-up period in all pairs of the variables in the two modalities (Table 4). Recoveries in the supply level of clean tap water and toilet hygiene correlated with a decreased prevalence of GIS. Dot plots of changes (Δ) in the prevalence of GIS in each shelter, divided by improvement in the level of tap water supply or toilet hygiene, are shown in Figure 7. Both restored tap water supply (p < 0.0001) and toilet hygiene (p = 0.005) were significantly associated with a decreased prevalence of GIS after the follow-up period. Meanwhile, a change in the food supply level was positively correlated with a change in the prevalence of fever, which may be partly explained by the delayed impact (i.e., several days later) of scarce resource supply on subsequent morbidity among evacuees, as described in section 3.3 below.

**Table 3:** Correlation coefficients (rho) between resource supply level and concurrent health status at the two cross-sectional assessment periods.

First cross-sectional assessment period (days 14–19)				
	*Health status*			
	All patients (%)	Fever ≥38.0 °C (%)	URS (%)	GIS (%)
Bottled water	−0.007 (p = 0.973)	+0.271 (p = 0.164)	−0.032 (p = 0.871)	−0.263 (p = 0.177)
Tap water	−0.275 (p = 0.157)	−0.196 (p = 0.319)	−0.335 (p = 0.081)	−0.376 (p = 0.049)
Food	+0.311 (p = 0.107)	+0.194 (p = 0.324)	−0.139 (p = 0.481)	−0.125 (p = 0.527)
Electricity	−0.108 (p = 0.584)	+0.370 (p = 0.053)	−0.095 (p = 0.630)	+0.067 (p = 0.734)
Blanket	−0.011 (p = 0.955)	+0.151 (p = 0.444)	+0.099 (p = 0.617)	+0.016 (p = 0.934)
Heating	+0.370 (p = 0.053)	−0.219 (p = 0.263)	+0.073 (p = 0.712)	−0.183 (p = 0.351)
Toilet hygiene	−0.048 (p = 0.807)	−0.461 (p = 0.014)	−0.242 (p = 0.215)	−0.509 ∗ (p = 0.006)
Second cross-sectional assessment period (days 20–25)				
Bottled water	−0.599 ∗∗ (p < 0.001)	−0.044 (p = 0.825)	−0.448 (p = 0.017)	−0.146 (p = 0.457)
Tap water	−0.563 ∗ (p = 0.002)	+0.070 (p = 0.723)	−0.643 ∗ (p < 0.001)	−0.175 (p = 0.372)
Food	−0.450 (p = 0.016)	+0.045 (p = 0.821)	−0.355 (p = 0.064)	−0.256 (p = 0.188)
Electricity	−0.401 (p = 0.034)	+0.194 (p = 0.322)	−0.528 ∗ (p = 0.004)	−0.011 (p = 0.954)
Blanket	+0.003 (p = 0.989)	−0.025 (p = 0.899)	−0.071 (p = 0.721)	+0.106 (p = 0.593)
Heating	−0.156 (p = 0.429)	−0.093 (p = 0.639)	−0.133 (p = 0.500)	−0.499 ∗ (p = 0.007)
Toilet hygiene	−0.297 (p = 0.125)	−0.047 (p = 0.813)	−0.366 (p = 0.055)	−0.335 (p = 0.082)

GIS, gastrointestinal symptoms; URS, upper respiratory symptoms.

The Spearman's correlation coefficients (rho) between the cross-sectional resource supply level and symptom prevalence among the enrolled 28 shelters are shown in this correlation matrix. The upper half is the correlation matrix in the 1^st^ assessment period, and the lower half is that in the 2^nd^ assessment period. The shown p-value aside of each correlation coefficient is the result of the test of no correlation.

∗p < 0.01; ∗∗p < 0.001.

**Table 4:** Correlation coefficients (rho) between changes in resource level and symptom prevalence.

		Changes (Δ) in the prevalence of symptoms			
		All patients (%)	Fever ≥38.0 °C (%)	URS (%)	GIS (%)
Changes (Δ) in resource supply	Bottled water	−0.129 (p = 0.515)	+0.173 (p = 0.380)	+0.249 (p = 0.201)	+0.159 (p = 0.419)
	Tap water	+0.042 (p = 0.832)	−0.194 (p = 0.323)	−0.070 (p = 0.722)	−0.506 ∗(p = 0.006)
	Food	+0.304 (p = 0.116)	+0.491 ∗(p = 0.008)	+0.101 (p = 0.610)	+0.179 (p = 0.361)
	Electricity	−0.055 (p = 0.781)	+0.203 (p = 0.300)	−0.015 (p = 0.940)	−0.016 (p = 0.938)
	Blanket	+0.036 (p = 0.856)	+0.236 (p = 0.227)	+0.192 (p = 0.328)	−0.003 (p = 0.989)
	Heating	−0.009 (p = 0.965)	+0.020 (p = 0.919)	−0.081 (p = 0.681)	−0.076 (p = 0.700)
	Toilet hygiene	−0.018 (p = 0.928)	−0.184 (p = 0.349)	−0.093 (p = 0.638)	−0.484 ∗(p = 0.009)
Change in population density		−0.267 (p = 0.169)	−0.118 (p = 0.550)	+0.001 (p = 0.996)	+0.321 (p = 0.096)

GIS, gastrointestinal symptoms; URS, upper respiratory symptoms.

The Spearman's correlation coefficients (rho) between the change in resource level and change in symptom prevalence among the enrolled 28 shelters are shown in this correlation matrix. The shown p-value aside of each correlation coefficient is the result of the test of no correlation.

∗p < 0.01.

### 3.3 The delayed impact of resource supply levels on health status

To clarify the delayed impact of the resource supply level on the subsequent (three to nine days later) health statuses of the evacuees in each shelter, the correlation coefficients between the resource supply levels in the cross-sectional analysis and the subsequent prevalence of common diseases at the follow-up analysis were calculated (Table 5). The supply level of clean tap water significantly affected the subsequent rate of patients (rho = −0.62, p = 0.0005) and the prevalence of URS (rho = −0.62, p = 0.0005). Although it did not reach statistical significance, the food supply level showed a weak negative correlation with the subsequent prevalence of fever after several days or a week (rho = −0.36, p = 0.0595). Together with the rapid recovery and fluctuations in food supply levels (data not shown), this delayed impact on the subsequent prevalence of fever may have produced an apparent positive correlation between changes in food supply and fever prevalence, as shown in section 3.2.

**Table 5:** Correlation coefficients (rho) between preceding resource levels and subsequent morbidity.

		Follow-up assessment period (i.e., 3–9 days later)			
		All patients (%)	Fever ≥38.0 °C (%)	URS (%)	GIS (%)
Initial assessment period (days 14–19)	Bottled water	−0.250 (p = 0.199)	+0.048 (p = 0.808)	−0.217 (p = 0.268)	−0.250 (p = 0.200)
	Tap water	−0.616 ∗∗(p < 0.001)	+0.016 (p = 0.936)	−0.616 ∗∗(p < 0.001)	−0.186 (p = 0.344)
	Food	−0.059 (p = 0.766)	−0.361 (p = 0.060)	−0.221 (p = 0.258)	−0.066 (p = 0.738)
	Electricity	−0.258 (p = 0.184)	+0.289 (p = 0.136)	−0.259 (p = 0.183)	−0.026 (p = 0.897)
	Blanket	+0.136 (p = 0.492)	+0.110 (p = 0.579)	−0.081 (p = 0.682)	−0.003 (p = 0.987)
	Heating	0.350 (p = 0.068)	−0.079 (p = 0.691)	+0.143 (p = 0.468)	−0.222 (p = 0.257)
	Toilet hygiene	−0.372 (p = 0.051)	−0.234 (p = 0.230)	−0.313 (p = 0.105)	−0.224 (p = 0.253)

GIS, gastrointestinal symptoms; URS, upper respiratory symptoms.

To evaluate the delayed impact of resource supply level on the health status, Spearman's correlation coefficients (rho) between the resource supply level in the first assessment period and subsequent symptom prevalence in the second assessment period among the enrolled 28 shelters are calculated. The shown p-value aside of each correlation coefficient is the result of the test of no correlation.

∗∗p < 0.001.

### 3.4 Correlation networks

**Figure 8:**
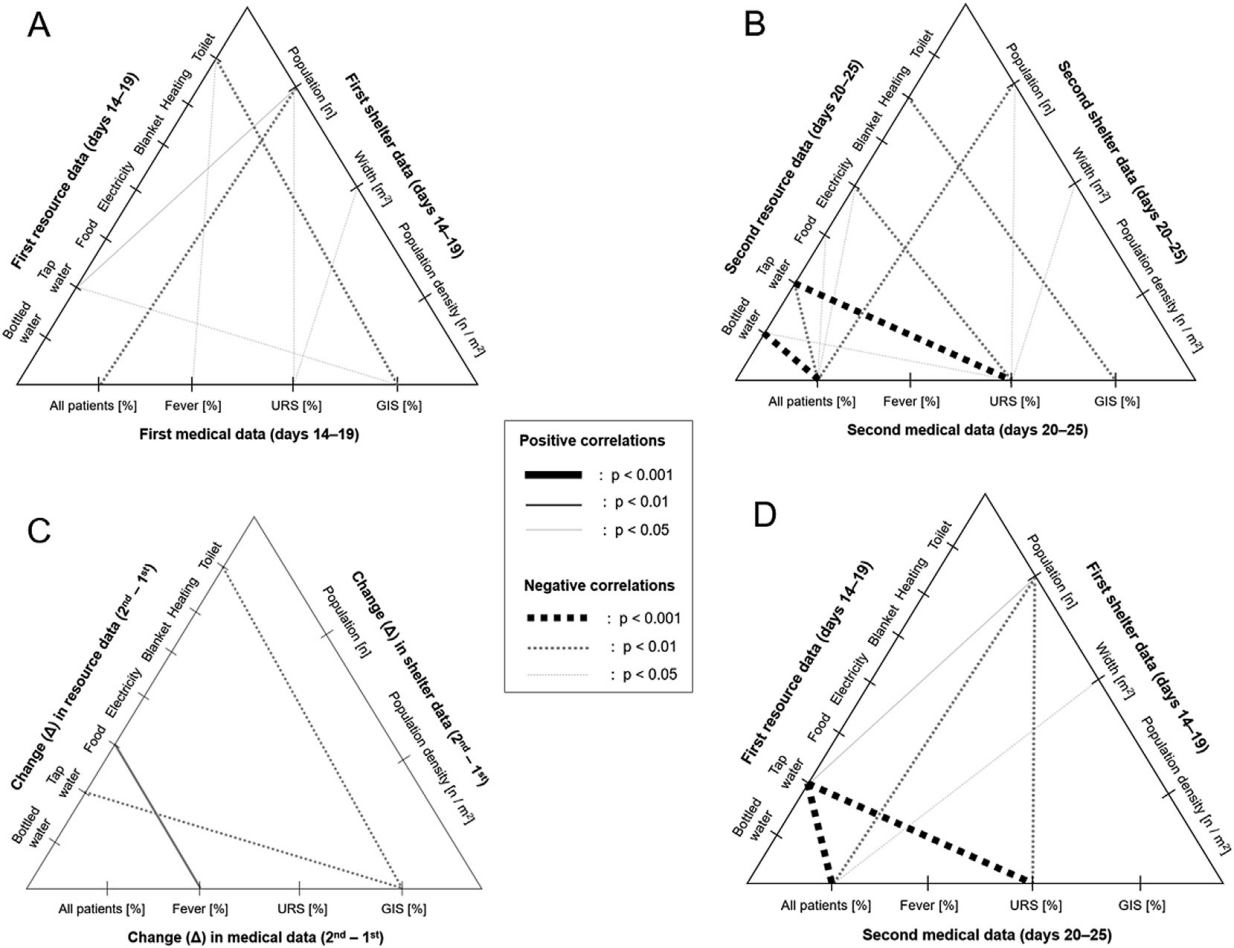
Correlation networks between resource supply levels, shelter sizes, and health status. (A) Correlation networks between resource supply levels and concurrent health statuses of the evacuees in each shelter during the first assessment period. (B) The same during the second assessment period. (C) Correlation networks between the changes (Δ) in resource supply level and in health status. (D) Correlation networks between resource supply levels during the first assessment period and delayed health statuses during the second assessment period in each shelter. GIS, gastrointestinal symptoms; URS, upper respiratory symptoms.

Based on the aforementioned results, the correlation networks between the resource supply levels, shelter sizes, and health statuses with concurrent timing and time lag are shown in Figure 8. Among the studied resources, clean tap water supply and toilet hygiene were suggested to be important for maintaining a high standard of health conditions in evacuation shelters. Notably, clean tap water supply showed significant correlations with at least one of the evaluated medical assessment data in all networks with different timings, which implied that it was a highly important factor in preventing common diseases in the shelters.

### 3.5 Relationship between restorations in tap water supply and in toilet hygiene

Based on the results that both the clean tap water supply and toilet hygiene were important to suppress environmental health risk among evacuees, we determined the correlations between the evaluated resource levels. During the first assessment period, only the tap water and toilet hygiene pair showed a significantly positive correlation (rho = +0.616, p = 0.0005), which was confirmed again in the second assessment period (rho = +0.744, p < 0.0001). Meanwhile, when evaluated by the changes (Δ) between the two assessment periods, $\Delta_{tap\;water}$ and $\Delta_{toilet\;hygiene}$ showed only a weak, non-significantly positive correlation (rho = +0.332, p = 0.0848).

To confirm whether the observed relationship between the restored toilet hygiene and decreased prevalence of GIS was independent of the restored tap water supply, the non-parametric partial correlation coefficient between $\Delta_{toilet\;hygiene}$ and $\Delta_{GIS}$, controlled for $\Delta_{tap\;water}$, was calculated. The calculated partial correlation coefficient was −0.389 (p = 0.045). For reference, the non-parametric partial correlation coefficient between $\Delta_{tap\;water}$ and $\Delta_{GIS}$, controlled for $\Delta_{toilet\;hygiene}$, was −0.418 (p = 0.030). These results suggest that, although both the clean tap water supply and toilet hygiene may have impact on the subsequent health status in the shelters, their supply levels were closely associated with each other and may have confounded the association between $\Delta_{toilet\;hygiene}$ and $\Delta_{GIS}$.

### 3.6 Evaluation of the shelter size-based bias

It is known that too many evacuees to a limited number of toilets would disturb the hygiene level in the toilets [1]. To reject the possible existence of bias from the shelter size to the toilet hygiene, we evaluated the Spearman correlation coefficient between shelter size (number of evacuees) and concurrent toilet hygiene level both in the first and second assessment periods. The calculated correlation coefficients in the first (rho = +0.135, p = 0.4931) and second (rho = +0.022, p = 0.9111) periods, were not statistically significant.

Next, to reject the possible existence of bias from the shelter size to the observed resource-health relationship among evacuees, we rechecked the correlation between resource recovery ($\Delta_{tap\;water}$ or $\Delta_{toilet\;hygiene}$) and decreased prevalence of GIS ($\Delta_{GIS}$) after stratifying shelters by size into ≥300 (14 shelters) and <300 (14 shelters) evacuees. The correlation coefficients between $\Delta_{tap\;water}$ and $\Delta_{GIS}$ were rho = −0.493 (p = 0.0730) and rho = −0.570 (p = 0.0335) in shelters with ≥300 and <300 evacuees, respectively. The correlation coefficients between $\Delta_{toilet\;hygiene}$ and $\Delta_{GIS}$ were rho = −0.360 (p = 0.2061) and rho = −0.596 (p = 0.0247) in shelters with ≥300 and <300 evacuees, respectively. Although all the correlation coefficients did not reach statistical significance level because of the small number of shelters after stratification, both pairs showed negative correlations even after stratification by shelter size, suggesting that bias from the shelter size was unlikely in this study.

## 4 Discussion

In this study, the correlation between the three modalities of evacuation including shelter size, resource supply level, and health situation in post-disaster evacuation shelters were cross-sectionally evaluated after the GEJE. This study was the first to systematically and continuously evaluate the conditions of multiple evacuation shelters after a devastating large-scale disaster. Although the enrolled shelters with complete eligible data were only 28 in this study, the observed prevalence rates of respiratory and digestive symptoms were similar to those of another previous report with different shelter cohorts after the GEJE [31]. The previous report supports the representativeness of the 28 shelters in this study concerning the health status assessment. This study delineated the difficulty and the importance of a rapid need assessment for resource supply in each post-disaster evacuation shelter to implement effective humanitarian actions for disaster victims in the acute phase of disasters with limited time and human resources. A shelter resource inspection system using quick visual assessment of resources, using morbidity among evacuees as the outcome factor, was implied to have validity.

The achieved assessment data demonstrated that resource supply levels significantly correlated with the concurrent and subsequent health status of the evacuees in each shelter. Restored clean tap water supply significantly correlated with a decrease in the prevalence of respiratory and gastrointestinal infectious conditions in evacuation shelters. The supply level of bottled clean water also showed a similar tendency in the second cross-sectional assessment period, but with weaker significance. The hygiene levels of the toilets also correlated with the medical situation in each shelter both cross-sectionally and longitudinally. Meanwhile, the population density in each shelter did not significantly correlate with the prevalence of common diseases in each shelter. In the cross-sectional analysis at the follow-up period (i.e., days 20–25), the electricity and heating supply levels were significantly correlated with the concurrent prevalence of common symptoms (second half of Table 3), but the significant effect of these resources disappeared in subsequent longitudinal analysis that evaluated the correlation between the changes in the studied variables. Collectively, the levels of clean water supply and toilet hygiene were implied to collaboratively decrease the environmental health risks in the shelters both cross-sectionally and longitudinally. The exact mechanism how improved clean tap water supply suppressed disease prevalence, which seems to be multifactorial, was not determined from this study. Conceivable theories include an increased opportunity of hand washing, tooth brushing, floor cleaning of living space, and toilet cleaning/flushing by using the restored water supply. A future research is needed to conclude the exact mechanism how a restoration of water supply suppress morbidity among the shelter evacuees.

That this study was done well before the 2019 worldwide coronavirus disease (COVID-19) pandemic, which continues till date, is important [32, 33]. Case isolation and the avoidance of areas with high population densities are theoretically and empirically suggested to be useful in decreasing the risk of infection with such highly transmittable pathogens [34, 35, 36, 37]. In the present study after the GEJE, where there was no such pandemic involving highly infectious pathogens, the population density in each shelter did not significantly affect the prevalence of common diseases. This could be different if the COVID-19 pandemic occurred after the GEJE. It should be emphasized that the results of this study do not deny the usefulness of social distancing in the face of a pandemic of highly infectious pathogens.

Lastly, the results of this study do not deny the importance of restoring resources other than clean water and toilet after massive disasters. Undoubtedly, both food and water are indispensable resources for all people with high priority for survival. A conceivable reason for the finding that changes (Δ) in food or electricity supply did not significantly correlate with the subsequent changes (Δ) in morbidity would be that the supply of these resources other than water supply and toilet hygiene were not so severely damaged in most of the enrolled 28 shelters, as shown in Figure 6. Consequently, evaluating the correlations between changes in resources and morbidity for these relatively preserved resources would not be an appropriate way to investigate the impact of restoring these resources after massive disasters on the environmental health. Cross-sectionally, electricity and heating supplies showed significant negative correlations with at least one of the concurrent medical assessment data, as shown in Figure 8B. This may imply the potential role of electricity supply in suppressing the risks of infectious diseases among the evacuees. The pattern of resource supply damage is largely influenced by the nature (type, scale, and location) of the disaster [38, 39]. To swiftly and effectively grasp the rapid need situations among evacuees, active surveillance by directly visiting the disaster-hit areas, interviewing the victims, and objectively assessing the resource supply levels will be desirable after each mass disaster.

There are several limitations to consider in reference to this study. First, small-sized shelters with <50 accommodated evacuees were not cross-sectionally assessed in this study. However, there were many disaster victims who evacuated to their cars or to their acquaintances’ homes. From the viewpoint of humanitarian aid, evacuees outside shelters should not be overlooked. Whether the observed findings on the epidemiological importance of resource supplies, especially clean tap water and toilet hygiene, to maintain a high standard health status among evacuees, are generalized to small shelters and off-shelter evacuees needs to be evaluated in future research. Moreover, the assessment data from 103 of the 131 mid-to-large-sized shelters (78.6%) could not be utilized in this study because of incomplete or sparse data. Such incomplete or missing data of essential variables need to be avoided as much as possible in future field surveillance after disasters. Another limitation was that the supply of hygienic face masks or rubbing of alcohol was not assessed in this study. The prevalence of face masks and practice of hand hygiene with rubbing of alcohol are effective for suppressing the spread of many infectious agents [40, 41]. Future research assessing the resource supply in evacuation shelters should also assess the dissemination of face masks and rubbing of alcohol. Further, assessment of the supply of soaps or detergents for laundry washing could be also helpful in future research. In humanitarian actions, not only the infrastructural or material resources but also human resources are important. The presence of persons in charge of health matters in each shelter was previously reported to possibly influence the sanitation level and environmental health status in the shelters, which may be better to be assessed in future research [42]. Another limitation was that the categorization system of the resource supply in this study was rough and relatively subjective, with only four steps (none/very bad, insufficient, sufficient, and excellent) of the supply levels. This could be one of the reasons why none of the evaluated resource supply levels showed statistically significant recovery between the two assessment periods. More precise and objective categorization systems that can be uniformly used worldwide, such as proclaimed in the Sphere Project [43], may be required in future research to evaluate the supply levels of food and water. Another limitation was that the detailed composition of personal attributes (composition of age, gender, single/household, or chronic physical and mental conditions) of the evacuees in each shelter was not evaluated in this study. The health status of a population depends largely on its population structure. Since the information would be relevant, it should be taken into consideration as a confounding factor in future research. Lastly, because of the relatively small number of enrolled shelters, we could not perform multivariate analysis including multiple regression analysis, since this would require sufficient sample size on relevant explanatory variables. To perform multivariate analyses, a greater number of eligible shelters will be required.

## 5 Conclusion

The present study delineated the difficulty and the importance of a rapid need assessment for resource supply to implement effective humanitarian actions for disaster victims in the acute phase of disasters with limited time and human resources. A shelter toilet inspection system using quick visual assessment has validity, if considered with water supply as a covariate and morbidity as the outcome factor. Developing methods for rapidly assessing, restoring, and equitably distributing the resources is an urgent need to prepare for massive catastrophic disasters in the future.

## Declarations

### Author contribution statement

Tetsuya Akaishi and Tadashi Ishii: Conceived and designed the experiments; Performed the experiments; Analyzed and interpreted the data; Contributed reagents, materials, analysis tools or data; Wrote the paper.

Kazuma Morino, Yoshikazu Maruyama and Satoru Ishibashi: Conceived and designed the experiments; Performed the experiments; Contributed reagents, materials, analysis tools or data.

Shin Takayama, Michiaki Abe, Takeshi Kanno and Yasunori Tadano: Analyzed and interpreted the data.

### Funding statement

This work was supported by the Japan Ministry of Education, Culture, Sports, Science and Technology (award number 1041554).

### Data availability statement

Data included in article/supplementary material/referenced in article.

### Declaration of interests statement

The authors declare no conflict of interest.

### Additional information

No additional information is available for this paper.

## Appendix A Supplementary data

Supplementary table.xlsx Supplementary Table 1. Raw data of resource supply and health status in the enrolled shelters. Data of the first assessment period were obtained during days 14–19, and data of the second assessment period were obtained during days 20–25 after the earthquake. GIS, gastrointestinal symptoms; Pt, patients. Supplementary table.xlsx
